# Minimum Information About a Simulation Experiment (MIASE)

**DOI:** 10.1371/journal.pcbi.1001122

**Published:** 2011-04-28

**Authors:** Dagmar Waltemath, Richard Adams, Daniel A. Beard, Frank T. Bergmann, Upinder S. Bhalla, Randall Britten, Vijayalakshmi Chelliah, Michael T. Cooling, Jonathan Cooper, Edmund J. Crampin, Alan Garny, Stefan Hoops, Michael Hucka, Peter Hunter, Edda Klipp, Camille Laibe, Andrew K. Miller, Ion Moraru, David Nickerson, Poul Nielsen, Macha Nikolski, Sven Sahle, Herbert M. Sauro, Henning Schmidt, Jacky L. Snoep, Dominic Tolle, Olaf Wolkenhauer, Nicolas Le Novère

**Affiliations:** 1 Database and Information Systems, Graduate Research School dIEM oSiRiS, Rostock University, Rostock, Mecklenburg-Vorpommern, Germany; 2 Centre for Systems Biology at Edinburgh, University of Edinburgh, Edinburgh, United Kingdom; 3 Informatics Life-Sciences Institute, School of Informatics, University of Edinburgh, Edinburgh, United Kingdom; 4 Biotechnology and Bioengineering Center, Department of Physiology, Medical College of Wisconsin, Milwaukee, Wisconsin, United States of America; 5 Department of Bioengineering, University of Washington, Seattle, Washington, United States of America; 6 Keck Graduate Institute, Claremont, California, United States of America; 7 National Centre for Biological Sciences, Tata Institute of Fundamental Research, Bangalore, India; 8 Auckland Bioengineering Institute, The University of Auckland, Auckland, New Zealand; 9 EMBL-EBI, Wellcome-Trust Genome Campus, Hinxton, United Kingdom; 10 Oxford University Computing Laboratory, University of Oxford, Oxford, United Kingdom; 11 Cardiac Electrophysiology Group, Department of Physiology, Anatomy and Genetics, University of Oxford, Oxford, United Kingdom; 12 Virginia Bioinformatics Institute, Virginia Polytechnic Institute and State University, Blacksburgh, Virginia, United States of America; 13 Engineering and Applied Science, The California Institute of Technology, Pasadena, California, United States of America; 14 Theoretical Biophysics, Humboldt Universität zu Berlin, Berlin, Germany; 15 Department of Cell Biology, University of Connecticut Health Center, Farmington, Connecticut, United States of America; 16 Laboratoire Bordelais de Recherche en Informatique, Universite Bordeaux 1, Bordeaux, France; 17 BIOQUANT, University of Heidelberg, Heidelberg, Germany; 18 Systems Biology & Bioinformatics Group, University of Rostock, Rostock, Germany; 19 Novartis Pharma AG, Novartis Campus, Basel, Switzerland; 20 Department of Biochemistry, Stellenbosch University, Matieland, South Africa; University of California San Diego, United States of America

Reproducibility of experiments is a basic requirement for science. Minimum Information (MI) guidelines have proved a helpful means of enabling reuse of existing work in modern biology. The Minimum Information Required in the Annotation of Models (MIRIAM) guidelines promote the exchange and reuse of biochemical computational models. However, information about a model alone is not sufficient to enable its efficient reuse in a computational setting. Advanced numerical algorithms and complex modeling workflows used in modern computational biology make reproduction of simulations difficult. It is therefore essential to define the core information necessary to perform simulations of those models. The Minimum Information About a Simulation Experiment (MIASE, Glossary in Box 1) describes the minimal set of information that must be provided to make the description of a simulation experiment available to others. It includes the list of models to use and their modifications, all the simulation procedures to apply and in which order, the processing of the raw numerical results, and the description of the final output. MIASE allows for the reproduction of any simulation experiment. The provision of this information, along with a set of required models, guarantees that the simulation experiment represents the intention of the original authors. Following MIASE guidelines will thus improve the quality of scientific reporting, and will also allow collaborative, more distributed efforts in computational modeling and simulation of biological processes.

**Box 1. Glossary**

**MIASE**

*Minimum Information About a Simulation Experiment*. Reporting guidelines specifying the information to be provided with the description of a simulation in order to permit its correct interpretation and reproduction.

**MIASE compliant**

A simulation description that provides all information listed by the MIASE guidelines.

**MIRIAM**

*Minimum Information Required in the Annotation of Models*. Reporting guidelines specifying the information to be provided with an encoded model in order to permit its correct interpretation and re-use.

**Model**

A mathematical representation of a biological system that can be manipulated and experimented upon (simulated).

**Model description**

Set of formal statements describing the structure of the components of a modeled system, whether entities or events, encoded in a computer-readable form.

**Repeatability**

The closeness between independent simulations performed with the same methods on identical models with the same experimental setup.

**Reproducibility**

The closeness between independent simulations performed with the same methods on identical models but with a different experimental setup.

**Simulation**

A numerical procedure performed on a model that aims to reproduce the spatial and temporal evolution (the behavior) of the system represented by the model, under prescribed conditions.

**Simulation experiment**

A set of procedures, including simulations, to be performed on a model or a group of models, in order to obtain a certain set of given numerical results.

## Needs for a Standard Description of Simulations Experiments

The rise of systems biology as a new paradigm of biological research has put computational modeling under the spotlight. In cell biology [1], physiology [2], and more recently in synthetic biology [3], mathematical modeling and simulation have become parts of a researcher's toolkit. Following Cellier [4], we consider “a model (M) for a system (S) and an experiment (E) is anything to which E can be applied in order to answer questions about S” and “a simulation is an experiment performed on a model”. Zeigler [5] emphasized the importance of separating the descriptions of the experimental frame (e.g., the initial conditions), the model, and the simulation.

Although generic, this framework for modeling and simulation applies well to the field of computational modeling and simulation of biological processes, where models are created and simulated as testable hypotheses in order to determine whether or not they are compatible with experimental data or expected future observations; their analysis supports the design of additional experiments and helps in the synthesis of engineered biological systems. The acceptance of the computationally aided systems biology approach has led to the creation of models at an ever increasing rate, as shown by the rapid growth of model databases. Because of the size of the systems considered, and their multi-scale aspects (both temporal and spatial), modeling activity in integrative systems biology requires researchers to leverage new approaches from prior work. Initiatives to establish standards for describing models and simulations have already been advocated in 1969, e.g., to “establish a standard form of what a model should be like, how it should be described and documented […]. This is intended in part to facilitate communication of information about models, which may be difficult owing to their complexity” [6].

Such an endeavor requires the model descriptions (specifying the mathematical expressions and parameters for a given model) to be stored and exchanged in a way that allows for their efficient reuse [7], [8]. Once the model descriptions are retrieved, the user typically wants to test existing simulation protocols on them to obtain a desired output. Currently, most users do so by reading the simulation description in the corresponding publication. This is, however, not only time-consuming, but also error prone. In some cases the published description of a simulation experiment is incomplete, or even wrong, and it requires educated guesswork to reconstruct the original experiment. Examples for such guesses include the initial conditions of simulation, the determination of a starting point for bifurcation diagrams, or the normalization of raw simulation output. Incomplete or erroneous descriptions impede reuse and replication of existing work, and hamper the use of models for educational purposes. Conversely, making this information available to others leads to a greater reuse of existing models.

Standardization plays a central role in facilitating the exchange and interpretation of the outcomes of scientific research, and in particular of computational modeling [9]. Defining which information must be provided when describing an experimental procedure is the task of reporting guidelines, federated in the global project Minimum Information for Biological and Biomedical Investigations (MIBBI) [10]. Those reporting guidelines generally result from consultations with a large community and are carefully thought out. To facilitate reuse of models, MIRIAM [11] was defined in 2005. MIRIAM is a set of rules describing the information that must be provided with a mathematical model in order to allow its effective reuse. Most of the MIRIAM rules deal with the origin and structure of the model, and the precise identification of its components. But the MIRIAM guidelines also state that:

The model, when instantiated within a suitable simulation environment, must be able to reproduce all relevant results given in the reference description that can readily be simulated.

While mentioning the need for result reproducibility, MIRIAM does not set out to cover the information needed to simulate the models.

As a consequence, it is still necessary to define the core information that needs to be made available to the users of existing models, so that they can perform defined simulations on those models. Once encoded in a computer readable format, these simulation experiment recipes can be downloaded along with the models, either from public resources or publisher Web sites. This will not only allow one to store descriptions of simulation experiments and reproduce them, but also foster their exchange between co-workers, research groups, and even between simulation tools. In this paper, we describe the minimum information that must be provided to make the description of a simulation experiment available to others. Experiment descriptions that provide all necessary information specified in the guidelines are considered MIASE compliant.

## Scope of MIASE

MIASE sets out to define minimum requirements for simulation descriptions. It covers the simulation procedures, and allows for the experiments to be reproduced. The particular focus of MIASE is on life science applications.

### MIASE Covers Simulation Procedures

One of the difficulties in applying common guidelines to multiple simulation methods is that the definitions of model and simulation vary, and there is an ill-defined line between the two concepts. This conceptual entanglement is sometimes at the core of mathematical and computational approaches, as with executable biology [12], where the model *is* the simulation algorithm itself. When the description of biological processes builds on numerical integration, there is often a clear conceptual distinction between a model definition and its numerical simulation over space and time. Both concepts are nevertheless sometimes merged at the level of the description formats. Experienced modelers use this feature to run advanced simulations that may even involve the combination of several models. However, for the purpose of the present discussion, the term “simulation” stands for any calculation performed on a model and describing evolutions of the biological system represented, for instance, over spatial and/or temporal dimensions. This includes, but is not limited to, time series simulations (describing the evolution of model variables over time), parameter scans (iterating a given simulation for a range of parameter combinations), sensitivity analyses (variation of parameters or other model properties according to some algorithm, with additional post-processing such as statistical analysis of results), and bifurcation analyses (experiments to study and find stable and unstable steady states). Every necessary piece of information contributing to the unambiguous description of such a simulation is part of the MIASE guidelines. Conversely, information required for the description of the model structure (covered by MIRIAM) for the determination of the model's parameterization, and the specifics of simulation experimental setups, are not part of the MIASE guidelines.

### MIASE Is a Reporting Guideline

Reporting guidelines describe how to report clearly and unambiguously what has been done, by describing the entities involved in the experiment. They are not, on the contrary, meant to describe which experimental approaches are correct, or how an experiment should be performed [13]. MIASE is a therefore neither a standard operating procedure nor a description of correct experimental approaches. As such, MIASE does not cover assumptions made during model design or simulation procedure. As mentioned above, information needed for the model description itself is listed in the MIRIAM guidelines. MIRIAM specifies the information necessary to correctly interpret the model, but does not require the explicit statement as to why this model was chosen to represent a particular biological process. Similarly, the reasons behind the choice of a particular simulation approach, e.g., using a stochastic rather than a deterministic algorithm, are not necessary for a MIASE-compliant simulation description. Also, MIASE does not require any statement about the correctness or the scope of a simulation experiment. Whether or not the simulation results match biological reality and whether or not an experiment should be conducted on a certain model is outside MIASE's mission. Nevertheless, a MIASE-compliant description should be detailed enough to allow others to investigate and discuss whether the experiment setup is correct.

### MIASE Enables the Reproduction on Different Experimental Setup

The scope of MIASE is limited to the *reproducibility* of the simulation experiment, rather than its *repeatability*. Reproducibility deals with the replication of experiments, possibly with a different simulation set up, such as using different simulation tools, while repeatability requires the possibility of replicating a simulation experiment on the same models within the very same simulation environment. Furthermore, MIASE's scope does not include the reproduction of identical numerical results of such an experiment. However, while MIASE does not deal with correctness of simulation results, we encourage modelers to provide means to check that the reproduced simulation experiment provides adequate results, e.g., by providing unique identifiers to the original result.

### MIASE Applies to Any Simulation Procedure in Life Science

The MIASE guidelines apply to simulation descriptions of biological systems that could be (but are not necessarily) written with ordinary and partial differential equations. For the time being, and as a consequence of the fact that the effort was launched in the systems biology community, the MIASE guidelines are applicable to the simulation of mathematical models of biochemical and physiological systems. However, MIASE principles are general and should appeal to other communities. It can be expected that MIASE compliance will be directly applicable to a wider range of simulation experiments, such as the ones performed in computational neuroscience or ecological modeling. MIASE could even be extended to cover other areas of mathematical modeling in the life sciences, e.g., process algebra.

## The MIASE Guidelines

MIASE is composed of rules, summarized in Box 2, that fall into three categories. Rules 1A to 1D list the information that must be provided about the models to be used in the simulation experiment. All models must be listed or described in a manner that enables the reproduction of the experiment. Rules 2A to 2D specify how to describe the simulation experiment itself. All information necessary to run any step of the experiment must be provided. Finally, rules 3A and 3B deal with the output returned from the experiment. A publication describing a simulation experiment must obey the three levels of rules for the description to be declared MIASE compliant. Detailed explanations of the rules and the rationale behind them is provided in Text S1, and also on the MIASE Web site (http://biomodels.net/miase/). Three examples showing the application of the MIASE rules are described in Text S2.

**Box 2. Rules for MIASE-Compliant Description of a Simulation Experiment**

1. All models used in the experiment must be identified, accessible, and fully described.
  - The description of the simulation experiment must be provided together with the models necessary for the experiment, or with a precise and unambiguous way of accessing those models.
  - The models required for the simulations must be provided with all governing equations, parameter values, and necessary conditions (initial state and/or boundary conditions).
  - If a model is not encoded in a standard format, then the model code must be made available to the user. If a model is not encoded in an open format or code, its full description must be provided, sufficient to re-implement it.
  - Any modification of a model (pre-processing) required before the execution of a step of the simulation experiment must be described.
2. A precise description of the simulation steps and other procedures used by the experiment must be provided.
  - All simulation steps must be clearly described, including the simulation algorithms to be used, the models on which to apply each simulation, the order of the simulation steps, and the data processing to be done between the simulation steps.
  - All information needed for the correct implementation of the necessary simulation steps must be included through precise descriptions or references to unambiguous information sources.
  - If a simulation step is performed using a computer program for which source code is not available, all information needed to reproduce the simulation, and not just repeat it, must be provided, including the algorithms used by the original software and any information necessary to implement them, such as the discretization and integration methods.
  - If it is known that a simulation step will produce different results when performed in a different simulation environment or on a different computational platform, an explanation must be given of how the model has to be run with the specified environment/platform in order to achieve the purpose of the experiment.
3. All information necessary to obtain the desired numerical results must be provided.
  - All post-processing steps applied on the raw numerical results of simulation steps in order to generate the final results have to be described in detail. That includes the identification of data to process, the order in which changes were applied, and also the nature of changes.
  - If the expected insights depend on the relation between different results, such as a plot of one against another, the results to be compared have to be specified.

## Conclusion and Perspectives

Biomedical sciences are witnessing the birth of a new era, comparable to physical engineering two centuries ago. The practice of systems biology, and its applied siblings synthetic biology and cell reprogramming, will require the use of modeling and simulations as a routine procedure. Investigations into the behavior of complex biological systems are increasingly predicated on comparing simulations to observations. The simulations must be reproduced and/or modified in controlled ways. Precise descriptions of the procedures involved is the first and mandatory step in any standardization effort.

Scientists involved in the simulation of biological processes at different scales and with different approaches, together with maintainers of standards in systems biology, developed MIASE through several physical meetings and online discussions (see http://biomodels.net/miase/). It is expected that such discussions will continue to develop as other life science communities join them. Efforts have been started to create software tools that can help users to apply MIASE rules. An example is the Simulation Experiment Description Markup Language (SED-ML; [14], http://biomodels.net/sed-ml/). Application programming interfaces are under development in various communities to facilitate the support of SED-ML by simulation tools.

The systematic application of MIASE rules will allow the reproduction of simulations, and therefore the verification of simulation results. Such transparency is necessary to evaluate the quality of scientific activity. It will also improve the sharing of simulation procedures and promotion of the collaborative development and use of models.

## Supporting Information

Text S1 Detailed description of the MIASE Guidelines, with a discussion of all the rules, and a workflow depicting the description of the different steps of a simulation experiment.
(0.19 MB PDF)

Text S2 Three examples of MIASE-compliant descriptions of different simulation experiments ran on the same model.
(0.48 MB PDF)
