# Supplementary material for: Minimum Information About a Simulation Experiment (MIASE)
Source: PLoS Comput Biol. 2011 Apr 28;7(4):e1001122. doi: 10.1371/journal.pcbi.1001122 (PMC3084216; doi:10.1371/journal.pcbi.1001122)

<u>Supporting information S1:</u> **Details on the MIASE Guidelines**

The following sections explain the conditions for a simulation experiment description to be MIASE compliant (summarized in Box 2 of the main text). A workflow for the setup of such a simulation description is given in Figure S1. Examples of MIASE compliant simulation descriptions are provided in supporting information S2.

Information about the models to use

An essential step is the precise specification of the model(s) used in the simulation experiment (**see Box 2 of the main text, Rule 1**). In order to be MIASE compliant, a simulation experiment description must identify any and all models used throughout the experiment. These models can be joined with the experiment description, or be made available via a link provided. If models are derived from existing models, the procedures used to derive them have to be precisely described (**Rule 1A**).

Simulation experiments need not be restricted to any one specific model; a simulation experiment description may apply to a number of models, possibly after minor adjustments. It is in fact expected that the same simulation steps may be run on different models, for instance to compare their behaviors, or to cope with model refinement. If, however, the experiment does not reference models, then a MIASE compliant description must instead provide access to a complete description of all of those models (Rule 1B-C). A model for which the code or the description are inaccessible, e.g. provided as a binary black box, does not allow a user and/or a software package to understand its structure and therefore to interpret fully the simulation experiment. This most often precludes the reproduction of the experiment (although in certain cases, with adequate information, it may not preclude its repeatability). As such closed models make exchange problematic or even futile, the MIASE guidelines strongly recommend usage of open machine-readable model descriptions. The use of models available in community-developed standard formats (such as the SBML [2], CellML [3] or NeuroML [4]) and complying with the MIRIAM guidelines is encouraged, when available and suitable.

If a model had previously been made publicly available, it should be referred to using a reference to that public resource. However, the reference must only lead to an unambiguously identifiable model. Other, less favored, possibilities include databases of models in non-standard formats, or reference to an actual implementation in source-code. MIASE compliance does not restrict the encoding of a model to particular specified formats.

It is often necessary to modify a model prior to simulation, e.g. certain model parameters may need refinement in order for the model to show a particular behavior during simulation. Apart from such simple modifications, models may undergo more complex procedures such as the replacement of a model constituent, whether entity, process or mathematics. These may be implicit and iterative, for instance in the case of a parameter scan. MIASE compliance demands changes to be clearly described within the simulation experiment description (**Rule 1D**). For the example of a parameter scan, the range over which the parameter shall be scanned and the sampling procedure must be provided in the description.

Information about the simulation steps

A MIASE compliant simulation experiment description must contain the information necessary to enable simulations to be run (**see Box 2, Rule 2**). This comprises the types of simulation, any relevant information specific to the simulation types, on which model(s) to apply which simulation

type(s), and in which order, and any other information necessary to reproduce a particular simulation run.

The simulation algorithms should be identified or referred to in an unambiguous way, taking into account the particular algorithm variants and their implementations (**Rule 2A**). This is essential, as different algorithms yield different numerical results for the same theoretical trajectory of the system. For example, integration schemes with polynomial interpolation schemes of a different order will yield different results, and implicit integration schemes may give different results than explicit schemes. The use of controlled vocabularies is recommended; for example, although work is at an early state, using terms from the Kinetic Simulation Algorithm Ontology (KiSAO, http://biomodels.net/kisao/). This facilitates the identification of similar algorithms in case the original cannot be readily re-used. Simulation workflows including sequential and nested simulation experiments must be described. If the simulation experiment is a sequence of different simulations run on different models and using intermediate results, possibly produced by different software, the exact order of the particular steps has to be clearly identified.

All information relevant to a particular simulation procedure must be provided (**Rule 2B**), including the aforementioned simulation algorithms, the range of values and sampling procedure in the case of parameter scans etc. For stochastic simulations, the random number generator and the number of repetitions should be provided. The meshing method used for discretization in some spatial simulations must be provided, although the description of the actual meshing is not covered by MIASE.

It may be that some or all of the simulation steps used for the original experiments were performed with closed-source simulation software, effectively black-boxes for which precise details of the simulation algorithms may be unknown, nor the details of their implementation. If so, all information necessary to reproduce the simulation steps, and not solely to repeat them (i.e. using the same "black box" approach), must be provided (**Rule 2C**). In effect this enables the re-implementation of the black box, so as to run the same simulation experiment. MIASE is designed to be used by researchers willing to exchange their simulation descriptions. A simulation procedure that is impossible to be fully understood and reproduced is not covered by MIASE. We recommend the information required for MIASE compliance be encoded in a standard description format, where such a format exists, so that existing tools can verify the faithful reproduction of simulation experiments. Examples of such standardization efforts are the *Simulation Experiment Description Markup Language* (SED-ML, [5]) or *CellML Metadata* [6].

Sometimes certain hardware or specific software libraries are required to produce correct results. For some types of experiments information about global simulation processes such as hybrid integrators or distributed compute jobs may also be needed. In such cases, MIASE-compliance demands an explanation of the use of that particular setting (**Rule 2D**). However, it must be pointed out that such information cannot be provided in a standard format for the time being, nor can the authors see a solution for it in the foreseeable future. It is nevertheless recommended to encode the explanation in natural language, until standard representations exist.

MIASE's rules are restricted to the parts of the simulation experiment *specific to the scientific problem*. Conversely, the influences that a particular system running the simulation has on the simulation outcome, such as the type of CPU or operating system, are outside the scope of MIASE. In particular all issues arising from real number equality (inconsistency in floating point arithmetic [7]) are not addressed by MIASE. Another example are the seeds used in stochastic simulations. These influences might lead to similar yet not identical simulation values. However, the variations are artifacts and the technical details underlying them are not considered *minimal* information. Nevertheless, even if this information is not *required* for MIASE compliance, its addition to the

simulation description is encouraged if it is essential, or even helpful for later use of the simulation experiment.

Information about the output

A simulation experiment produces a defined set of results, which is presented for the benefit of the end user, whether human or software. The production of these results is part of a MIASE compliant simulation experiment description (**see Box 2, Rule 3**).

It may be that the numerical results obtained from the simulation steps used in the experiment do not constitute the final desired output. A MIASE compliant experiment description must include all necessary procedures required to be applied to the raw simulation results in order to obtain the appropriate result (**Rule 3A**). Examples for such post-processing are the conversion of units from different simulation runs, normalization of results, or transformation of a trajectory into a movie.

The output of the simulation experiment can be presented under different forms, e.g. textual, in a table or using descriptors, but also graphical, or in a movie. While detailed characteristics of specific output types need not be specified, the general format to present results should be described (**Rule 3B**). A time-course, where some model variables are plotted against time provides different insights than a phase portrait that plots different model variables against one another. While MIASE covers the description of output types, it does not address the exact visual rendering of the simulation results. The visual description, such as the type and appearance of curves, movies, the scaling, or the labels, are not part of the minimal description, since this information is not necessary to understand and reproduce the simulation procedure. The same principle applies to the definition of output tables – while the process of gaining the data and specifying the content of the single columns is within the scope of MIASE, the specification of output formats, such as how to format numbers or the order of columns, is not considered relevant for MIASE compliance.

References

1. International Organisation for Standardisation (ISO). Information processing - Documentation symbols and conventions; program and system flowcharts. ISO 5807.
2. Hucka M, Finney A, Sauro HM, Bolouri H, Doyle JC et al (2003) The systems biology markup language (SBML): a medium for representation and exchange of biochemical network models. Bioinformatics 19: 524–531.
3. Cuellar AA, Lloyd CM, Nielsen PF, Bullivant DP, Nickerson DP et al. (2003) An Overview of CellML 1.1, a Biological Model Description Language. Simulation 79: 740-747.
4. Goddard NH, Hucka M, Howell F, Cornelis H, Shankar K et al. (2001) Towards NeuroML: model description methods for collaborative modelling in neuroscience. Philos. Trans. R. Soc. London, Ser. B 356: 1209-1228.
5. Köhn D, Le Novère N (2008). SED-ML - An XML Format for the Implementation of the MIASE Guidelines. Lecture Notes in Bioinformatics, 5307: 176-190.
6. Nickerson DP, Corrias A, Buist ML (2008) Reference descriptions of cellular electrophysiology models. Bioinformatics 24: 1112-1114.
7. Chesneaux J (1994) The equality relations in scientific computing. Numerical Algorithms 7: 129-143.

Figure S1

Flowchart representing the rules (see Box 2) for a MIASE compliant simulation. Rectangles \represent processes, diamonds represent decision points [1].

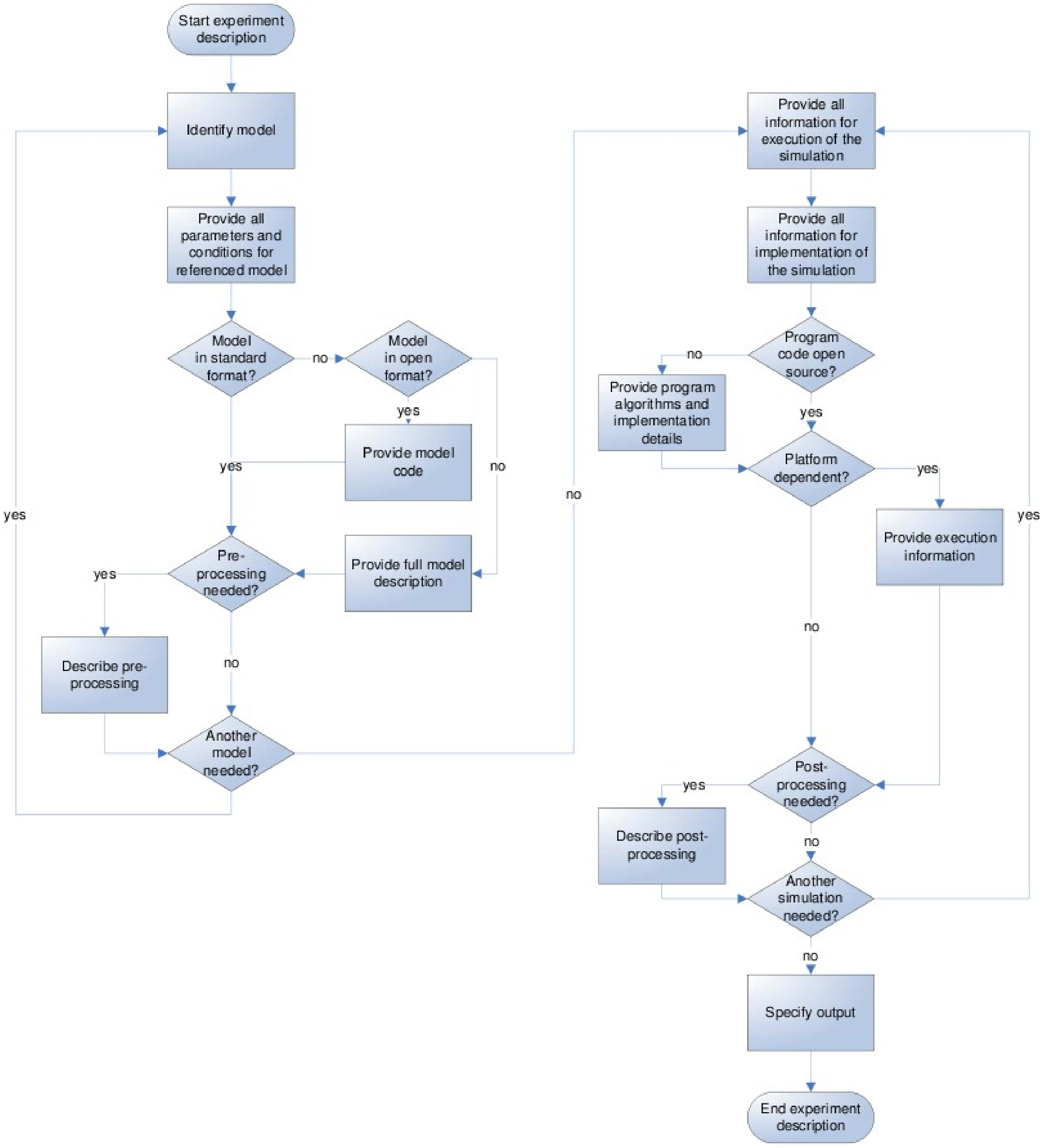

Supplement: Text S1 — Detailed description of the MIASE Guidelines, with a discussion of all the rules, and a workflow depicting the description of the different steps of a simulation experiment. (0.19 MB PDF) [file pcbi.1001122.s001.pdf]
