# Supplementary material for: Minimum Information About a Simulation Experiment (MIASE)
Source: PLoS Comput Biol. 2011 Apr 28;7(4):e1001122. doi: 10.1371/journal.pcbi.1001122 (PMC3084216; doi:10.1371/journal.pcbi.1001122)

Supporting text S2: **Examples of simulation experiments: Simulating the Repressilator**

The following examples are MIASE compliant descriptions of three simulation experiments performed on the well-known, simple Repressilator model with its rich and variable behavior. The Repressilator is a synthetic oscillating network of transcription regulators in Escherichia coli [1]. The network is composed of three repressor genes (lacI, tetR, and cI) and their promoters forming a cyclic negative-feedback loop. It is implemented in a simple mathematical model of coupled first-order differential equations which describe the interactions of the molecular species involved in the network. All six molecular species included in the network (three mRNAs, three repressor proteins) participate in creation (transcription/translation) and degradation processes. The model is used to determine the influence of the various parameters on the dynamic behavior of the system. In particular, parameter values were sought which would induce stable oscillations in the concentrations of the system components. Oscillations in the levels of the three repressor proteins are obtained by numerical integration.

The three introduced simulation experiments include a timecourse on the original model (**Example 1**), a timecourse on a perturbed version of the model (**Example 2**), and the creation of phase plane plot (**Example 3**).

Example 1

To run a time course simulation on the model as it is available from the BioModels Database [2], the following steps have to be followed (the execution will lead to Figure 1C of the original publication):

1. Import the model identified by the Unified Resource Identifier [3] "`urn:miriam:biomodels.db:BIOMD0000000012`" (NB: this is the reference for the model encoded in SBML and stored in BioModels Database. An equivalent reference for the model encoded in CellML and stored in the CellML repository [4] would be http://models.cellml.org/exposure/6ad4f33a31aa0b9aa81b6558979d72f5.) [**rules 1A+1B**]
2. Select a deterministic method "KISAO:0000035" (NB: this is the reference for a term of the Kinetic Simulation Algorithm Ontology) to run a simulation on that model. [**rule 2A**]
3. Run a uniform time course for the duration of 1000 minutes with an output interval of 1 min. [**rule 2B**]
4. Report the amount of Lactose Operon Repressor, Tetracycline Repressor and Repressor protein CI against time in a 2D Plot. [**rule 3B**]

The result of the simulation is shown in Figure S2.

Example 2

The fine-tuning of the model can be shown by changing parameters before simulation. The initial value of protein copies per promoter and leakiness in protein copies per promoter can be changed, which move the system's behavior from sustained oscillation to asymptotic steady-state. That change may be described as follows:

1. perform step 1 of Example 1.
2. Change the value of the parameter "tps_repr" from "0.0005" to "1.3e-05". [**rule 1D**]
3. Change the value of the parameter " tps_active" from "0.5" to "0.013". [**rule 1D**]

4. Select a deterministic method (KISAO:0000035) to run a simulation. [**rule 2A**]
5. Run a uniform time course for the duration of 1000 minutes with an output interval of 1 min. [**rule 2B**]
6. Report the amount of Lactose Operon Repressor, Tetracycline Repressor and Repressor protein CI against time in a 2D Plot. [**rule 3B**]

The result of the simulation is shown in Figure S3.

Example 3

The output of the simulation steps may be subjected to data post-processing before plotting it. In order to describe the production of normalized plots of the timecourse simulated above, representing the influence of one variable on another (in phase-planes), one would define the following steps.

1. Perform steps 1 to 3 of Example 1.
2. Collect the time series for lacI, tetR and cI, denoted as PX(t), PY(t) and (cI). [**rule 3A**]
3. Compute the value of the highest value for each of the repressor proteins, max(PX(t)), max(PY(t)), max(PZ(t)). [**rule 3A**]
4. Normalize the data for each of the repressor proteins by dividing each time point by the maximum value, i.e PX(t)/max(PX(t)), PY(t)/max(PY(t)) , and PZ(t)/max(PZ(t)). [**rule 3A**]
5. Report the normalized Lactose Operon Repressor protein as a function of the normalized Repressor protein CI, The normalized Repressor protein CI as a function of the normalized Tetracycline Repressor protein, and the normalized Tetracycline Repressor protein against the normalized Lactose Operon Repressor protein in a 2D plot. [**rule 3B**].

Figure S4 illustrates the result of the simulation after post-processing of the output data.

References

1. Elowitz MB, Leibler S (2000) A synthetic oscillatory network of transcriptional regulators. Nature 403: 335–338.
2. Le Novère N, Bornstein B, BroicherA, Courtot M, Donizello M et al. (2006) BioModels Database: a free, centralized database of curated, published, quantitative kinetic models of biochemical and cellular systems. Nucleic Acids Res 34: D689-D691.
3. Berners-Lee T, Fielding R, Masinter, L (2005) Uniform Resource Identifier (URI): Generic Syntax, http://www.ietf.org/rfc/rfc3986.txt
4. Lloyd CM, Lawson JR, Hunter PJ, Nielsen PF (2008) The CellML Model Repository. Bioinformatics 24: 2122-2123.
5. Hoops S, Sahle S, Lee C, Pahle J, Simus N et al. (2006) COPASI a complex pathway simulator. Bioinformatics 22: 3067–3074.

Figure S2

Time-course of the Repressilator model, imported from BioModels Database (BIOMD0000000012), simulated in COPASI [5], and plotted with Gnuplot (http://www.gnuplot.info/). The number of repressor proteins lacI, tetR and cI is shown as a function of the simulated time.

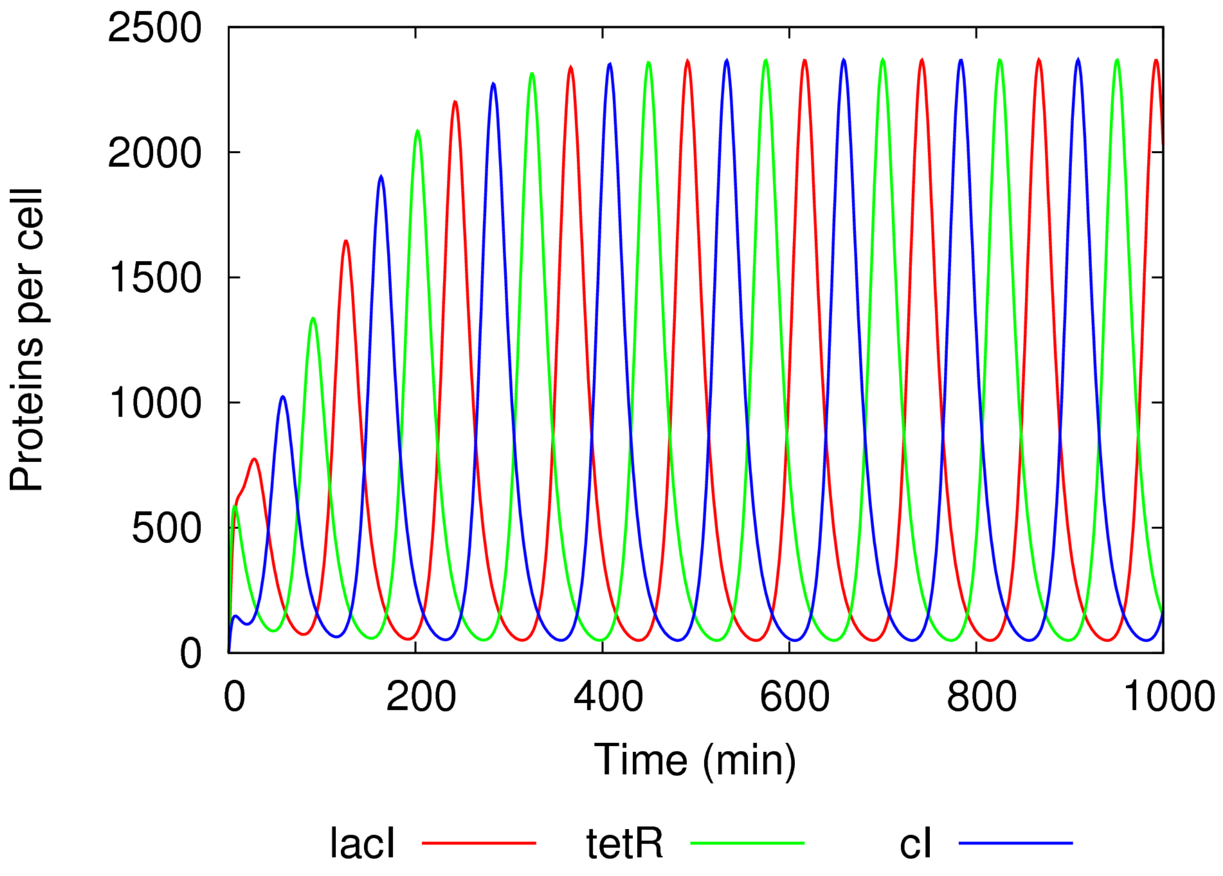

Figure S3

Timecourse of the Repressilator model, imported from BioModels Database (BIOMD0000000012), simulated in COPASI [29] after modification of the strength of the repressed and active promoters , and plotted with Gnuplot (http://www.gnuplot.info/). The number of repressor proteins lacI, tetR and cI is shown as a function of the simulated time.

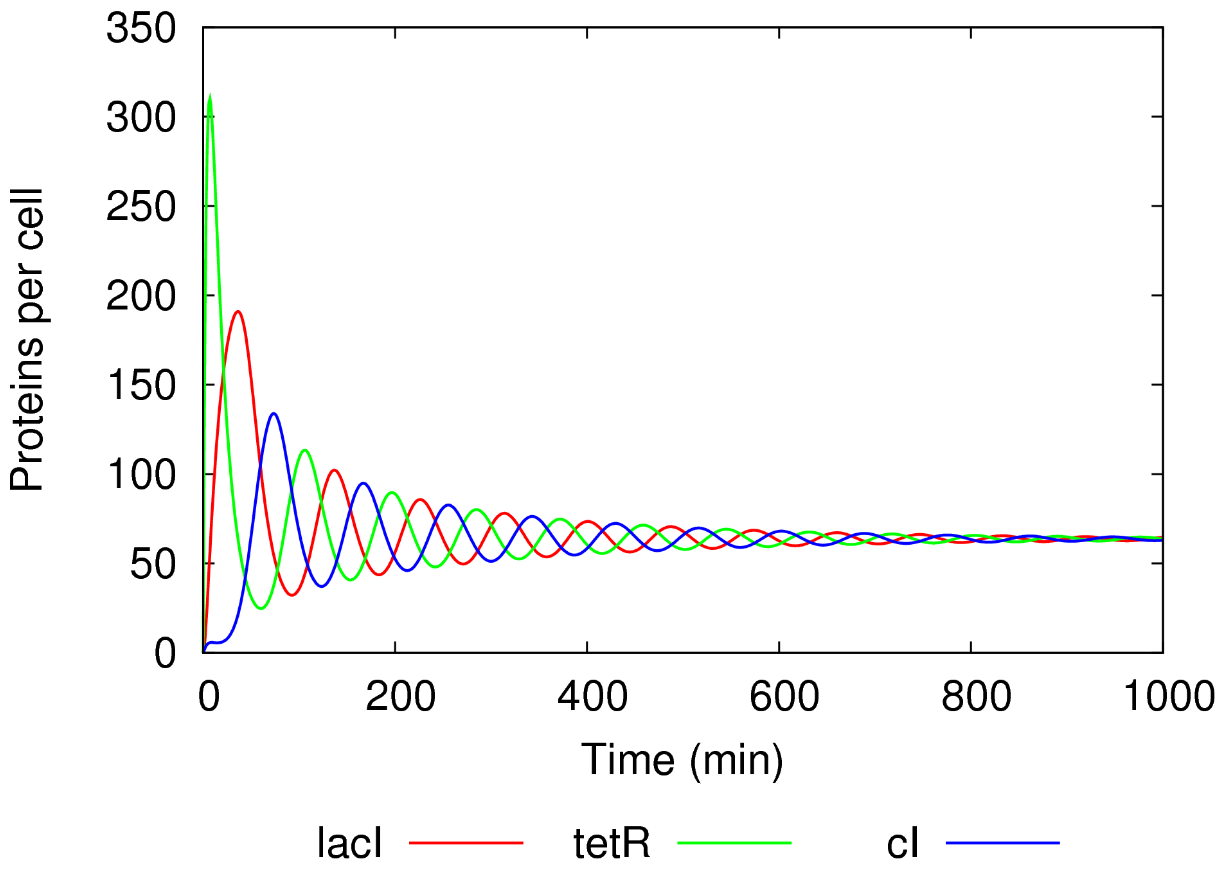

Figure S4

Timecourse of the Repressilator model, imported from BioModels Database (BIOMD0000000012), simulated in COPASI [29], and plotted with Gnuplot (http://www.gnuplot.info/), showing the normalized temporal evolution of repressor proteins lacI, tetR and cI in phase-plane.

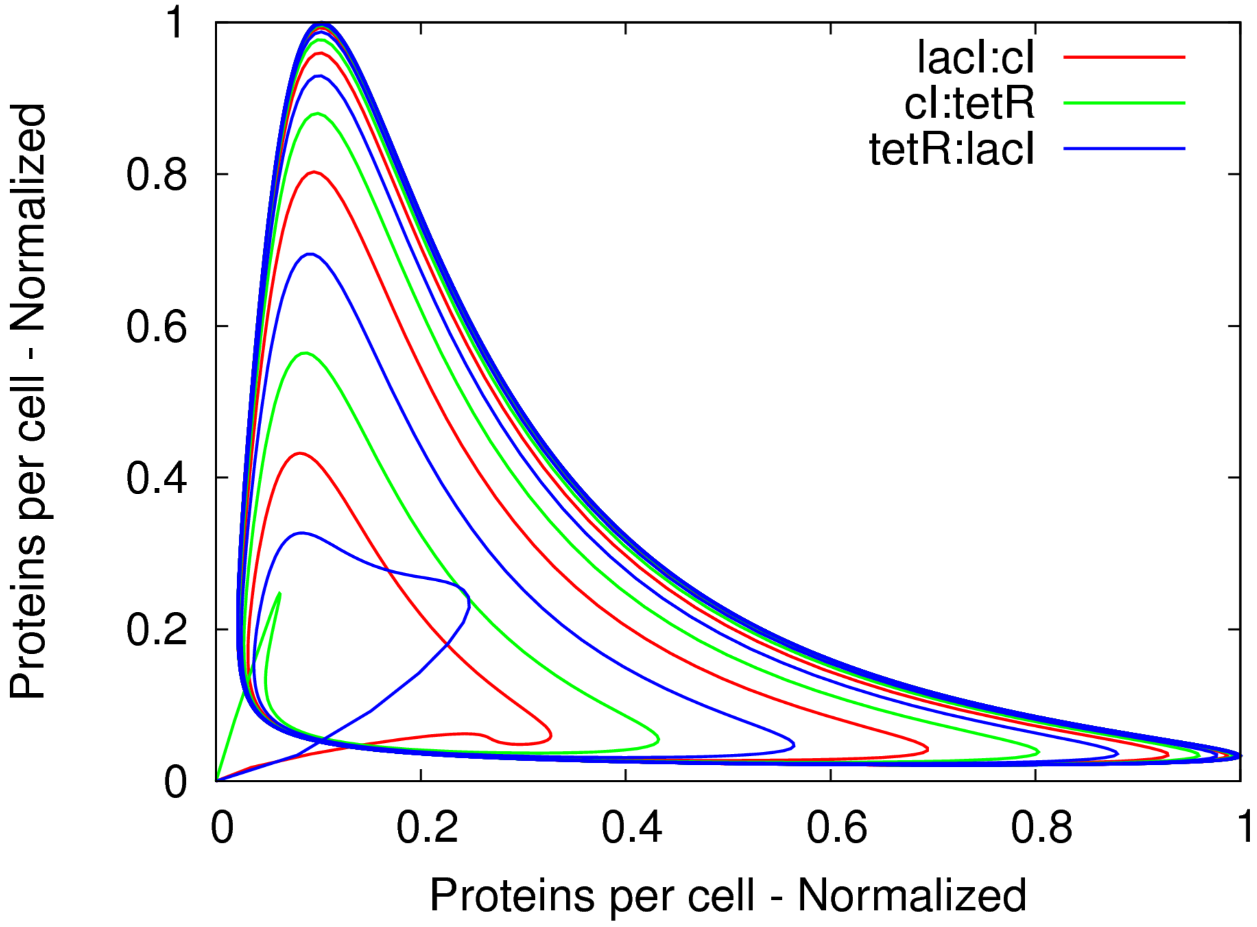

Supplement: Text S2 — Three examples of MIASE-compliant descriptions of different simulation experiments ran on the same model. (0.48 MB PDF) [file pcbi.1001122.s002.pdf]
